# High-Fat Diet-Induced Neuropathy of Prediabetes and Obesity: Effect of PMI-5011, an Ethanolic Extract of *Artemisia dracunculus* L.

**DOI:** 10.1155/2010/268547

**Published:** 2010-04-08

**Authors:** Pierre Watcho, Roman Stavniichuk, David M. Ribnicky, Ilya Raskin, Irina G. Obrosova

**Affiliations:** ^1^Pennington Biomedical Research Center, Louisiana State University System, Baton Rouge, LA 70808, USA; ^2^Biotech Center, Cook College, Rutgers University, New Brunswick, NJ 08901-8554, USA; ^3^Department of Nutritional Sciences, Cook College, Rutgers University, New Brunswick, NJ 08901-8554, USA

## Abstract

*Artemisia* species are a rich source of herbal remedies with antioxidant and anti-inflammatory properties. We evaluated PMI-5011, an ethanolic extract of *Artemisia dracunculus* L., on neuropathy in high-sfat diet-fed mice, a model of prediabetes and obesity developing oxidative stress and proinflammatory changes in peripheral nervous system. C57Bl6/J mice fed high-fat diet for 16 weeks developed obesity, moderate nonfasting hyperglycemia, nerve conduction deficit, thermal and mechanical hypoalgesia, and tactile allodynia. They displayed 12/15-lipoxygenase overexpression, 12(S)-hydroxyeicosatetraenoic acid accumulation, and nitrosative stress in peripheral nerve and spinal cord. PMI-5011 (500 mgkg^−1^d^−1^, 7 weeks) normalized glycemia, alleviated nerve conduction slowing and sensory neuropathy, and reduced 12/15-lipoxygenase upregulation and nitrated protein expression in peripheral nervous system. PMI-5011, a safe and nontoxic botanical extract, may find use in treatment of neuropathic changes at the earliest stage of disease.

## 1. Introduction

Diabetic distal symmetric sensorimotor polyneuropathy affects ~50% of patients with diabetes mellitus and is a leading cause of foot amputation [1]. Evidence for the development of neuropathic changes at the prediabetic stage, prior to development of overt hyperglycemia and diabetes mellitus, is emerging from both clinical [2–4] and experimental [5–7] studies. Except the aldose reductase inhibitor epalrestat in Japan and *α*-lipoic acid in several countries, no pathogenetic treatment for diabetic or prediabetic neuropathy is currently available. A number of pharmacological agents that showed promise in animal studies have been withdrawn from clinical trials because of a lack of efficacy or adverse side-effects [8, 9]. The development of nonpharmacological approaches and, among them, complementary and alternative medicines for prevention and treatment of diabetes and prediabetes-associated neuropathic changes is, therefore, highly warranted. 

The plant genus *Artemisia,* that includes over 1500 species, has been a rich source of herbal remedies in many countries [10, 11]. Several *Artemisia* species lower blood glucose concentrations and have been used for treatment of diabetes [12, 13]. Fluid extracts of *Artemisia* plant species have been reported to reduce oxidative stress associated with obesity [14] and to display anti-inflammatory and antinociceptive properties [15]. PMI-5011 [16, 17], an ethanolic extract of *Artemisia dracunculus L.* with a good safety profile [16], has been found to inhibit activity of aldose reductase [18], the first enzyme of the sorbitol pathway, known to play an important role in the pathogenesis of both diabetic and prediabetic neuropathy [6, 19–23]. 

We previously demonstrated that a high-fat diet (HFD)-fed mouse with alimentary obesity, hyperinsulinemia, and impaired glucose tolerance develops nerve conduction velocity deficit and small sensory fiber neuropathy and displays increased sorbitol pathway activity, oxidative-nitrosative stress, and pro-inflammatory changes in PNS. This mouse, therefore, represents an ideal model for evaluating PMI-5011 on functional and biochemical manifestations of prediabetic neuropathy. The findings reported herein provide evidence of alleviation of HFD-induced nerve conduction slowing and small sensory nerve fiber dysfunction after PMI-5011 treatment, potentially due to inhibition of oxidative-nitrosative stress and pro-inflammatory response in the peripheral nervous system (PNS).

## 2. Methods

### 2.1. Reagents

Unless otherwise stated, all chemicals were of reagent-grade quality, and were purchased from Sigma Chemical Co., St. Louis, MO. PMI-5011, an ethanolic extract of *Artemisia dracunculus L.*, was prepared and analyzed as described previously [17]. Ethanol was completely removed after extraction by heating in a rotavapor. Rabbit polyclonal (clone H-100) anti-12-lipoxygenase (LO) antibody was obtained from Santa Cruz Biotechnology, Santa Cruz, CA. Mouse monoclonal (clone 1A6) anti-nitrotyrosine (NT) antibody was purchased from Millipore, Billerica, MA. 

### 2.2. Animals

The experiments were performed in accordance with regulations specified by the Guide for the Care and Handling of Laboratory Animals (NIH Publication No. 85-23) and Pennington Biomedical Research Center Protocol for Animal Studies. Male C57Bl6/J mice, body weight ~23–25 g, were fed standard mouse chow (PMI Nutrition International, Brentwood, MO, USA) and had *ad libitum* access to water. After completion of body weight, non-fasting blood glucose and peripheral nerve function evaluation, the mice were randomly assigned to receive normal chow (NC) or HFD (D12328, 10.5 kcal% fat, and D 12330, 58 kcal% fat with corn starch, resp., Research Diets, Inc., New Brunswick, NJ), for 16 weeks (*n* = 33 per condition). Then body weights, non-fasting blood glucose concentrations, and variables of peripheral nerve function have been recorded again, and NC- and HFD-fed mice have randomly been divided into three matching in body weights and blood glucose concentrations subgroups. One subgroup has been euthanized for tissue harvest (*n* = 10 per condition). Two other subgroups of NC- and HFD-fed mice were maintained with (*n* = 13) or without (*n* = 10) treatment with PMI-5011 (500 mgkg^−1^d^−1^ in 2% Twin 80, 50 *μ*Lkg^−1^, by oral gavage), for another 7 weeks. After final body weight, blood glucose and peripheral nerve function measurements, all the mice have been euthanized. Throughout the study, non-fasting blood glucose measurements were performed from 9 till 11 am. Physiological and behavioral tests of peripheral nerve function have been done in the following order: tactile responses to flexible von Frey filaments (first day), thermal algesia by tail-flick test (second day), thermal algesia by paw withdrawal test (third day), mechanical algesia by Randall-Selitto test (fourth day), and motor (MNCV) and sensory (SNCV) nerve conduction velocities (fifth day). Measurements of MNCV and SNCV were taken in mice anaesthetized with a mixture of ketamine and xylazine (45 mgkg^−1^ body weight and 15 mgkg^−1^ body weight, resp., i.p.).

### 2.3. Anesthesia, Euthanasia, and Tissue Sampling

The animals were sedated by CO_2_, and immediately sacrificed by cervical dislocation. Sciatic nerves and spinal cords were rapidly dissected and frozen in liquid nitrogen for subsequent assessment of LO and nitrated protein expression and 12(S)hydroxyeicosatetraenoic acid [12(S)HETE] concentrations. The biochemical measurements have been performed before and after PMI-5011 treatment.

### 2.4. Specific Methods

#### 2.4.1. Physiological Tests

Sciatic MNCV and hind-limb digital SNCV have been measured as we have described elsewhere [24]. TCAT-2 Temperature Controller with RET-3 Temperature probe and HL-1, Heat Lamp (Physitemp Instruments, Inc., Clifton, NJ) was used to maintain body and hind-limb temperature at 37°*C*.

#### 2.4.2. Behavioral Tests

**(1) Tactile Responses**

Tactile responses were evaluated by quantifying the withdrawal threshold of the hindpaw in response to stimulation with flexible von Frey filaments. Mice were placed in individual plexiglass boxes on stainless steel mesh floor and were allowed to adjust for at least 20 minutes. A series of calibrated von Frey filaments (IITC Life Science, Woodland Hills, CA) was applied perpendicularly to the plantar surface of a hindpaw with sufficient force to bend the filament for 6 s. Typically, 6–8 mice were stimulated one after another, in the same order, during one testing procedure (~2-3 h), and stimulations were repeated 5-6 times. Brisk withdrawal or paw flinching was considered as a positive response. In the absence of a response in 50% or more paw stimulations, a filament of next greater force was applied. Stimulation was stopped at the filament producing a positive response in 4 out of 5 or 6 stimulations. The average value for buckling weights of the last and previous filaments (or weight of the last filament if the previous one(s) did not produce any responses) was considered a tactile response threshold, and was recorded for each paw. For example, if the buckling weight of the last filament producing 4 positive responses was 1.5 g, and stimulation with two previous filaments produced two positive responses to the 1.2 g filament and one positive response to the 1.0 g filament, then the tactile threshold would be [(1.5 × 4) + (1.2 × 2) + 1.0]/7 = 1.34 g. The means value was taken for statistical analysis.

**(2) Thermal Algesia**

**(a) Plantar (Hargreaves) Test**

To determine the sensitivity to noxious heat, mice were placed within a plexiglass chamber on a transparent glass surface and allowed to acclimate for at least 20 minutes. A thermal stimulation meter (IITC model 336 TG Combination Tail Flick and Paw algesia meter, IITC Life Science, Woodland Hills, CA) was used. The device was activated after placing the stimulator directly beneath the plantar surface of the hindpaw. The paw withdrawal latency in response to the radiant heat (15% intensity which produced a heating rate of ~1.3°C per s, cut-off time 30 s) was recorded. Floor temperature was set at ~32-33°C (manufacturer's setup). Individual measurements were repeated four to five times and the mean value calculated.

**(b) Tail-Flick Test**

For assessment of tail flick response latencies, the tail-flick and paw algesia meter described above was set at 40% heating intensity (heating rate ~2.5°C per s) with a cut-off at 10 s. Four to five readings per animal were taken at 15-minute interval, and the average was calculated.

**Mechanical Algesia**

Tail pressure thresholds were registered with the Paw/Tail Pressure Analgesia meter for the Randall-Selitto test (37215 - Analgesy-Meter, UGO-Basile, Comerio VA, Italy). Pressure increasing at a linear rate of 10 g with the cut-off of 250 g to avoid tissue injury was applied to the base of the tail. The applied tail pressure that evoked biting or licking behavior was registered by analgesia meter and expressed in g. Three tests separated by at least 15 minutes were performed for each animal, and the mean value of these tests was calculated.

#### 2.4.3. Biochemical Studies

**(1) Western Blot Analysis of LO and Nitrated Protein Expressions**

To assess LO and nitrated protein expressions by Western blot analysis, sciatic nerve and spinal cord materials (~20 mg) were placed on ice in 200 *μ*L of RIPA buffer containing 50 mmol/l Tris-HCl, pH 7.2; 150 mmol/l NaCl; 0.1% sodium dodecyl sulfate; 1% NP-40; 5 mmol/l EDTA; 1 mmol/l EGTA; 1% sodium deoxycholate and the protease/phosphatase inhibitors leupeptin (10 *μ*g/mL), pepstatin (1 *μ*g/mL), aprotinin (20 *μ*g/mL), benzamidine (10 mM), phenylmethylsulfonyl fluoride (1 mM), sodium orthovanadate (1 mmol/l), and homogenized on ice. The homogenates were sonicated (4 × 10 s) and centrifuged at 14,000 g for 20 minutes. All the aforementioned steps were performed at 4°C. The lysates (20 and 40 *μ*g protein for sciatic nerve and spinal cord, resp.) were mixed with equal volumes of 2× sample-loading buffer containing 62.5 mmol/l Tris-HCl, pH 6.8; 2% sodium dodecyl sulfate; 5%  *β*-mercaptoethanol; 10% glycerol and 0.025% bromophenol blue, and fractionated in 10% (nitrated proteins) or 7.5% (LO) sodium dodecyl sulfate polyacrylamide gel in an electrophoresis cell (Mini-Protean III; Bio-Rad Laboratories, Richmond, CA). Electrophoresis was conducted at 15 mA constant current for stacking, and at 25 mA for protein separation. Gel contents were electrotransferred (80 V, 2 h) to nitrocellulose membranes using Mini Trans-Blot cell (Bio-Rad Laboratories, Richmond, CA) and western transfer buffer (25 mmol/l Tris-HCl, pH 8.3; 192 mmol/l glycine; and 20% (v/v) methanol) [19]. Free binding sites were blocked in 2% and 5% (w/v) bovine serum albumin (for nitrated proteins and LO, resp.) in 20 mmol/l Tris-HCl buffer, pH 7.5, containing 150 mmol/l NaCl and 0.05% Tween 20, for 1 h, after which LO or nitrotyrosine antibodies were applied for 2 h, for detection of nitrated protein and LO expressions. The horseradish peroxidase-conjugated secondary antibody was then applied for 1 h. After extensive washing, protein bands detected by the antibodies were visualized with the Amersham ECL Western Blotting Detection Reagent (Little Chalfont, Buckinghamshire, UK). Membranes were then stripped in the 25 mmol/l glycine-HCl, pH 2.5 buffer containing 2% SDS, and reprobed with *β*-actin antibody to confirm equal protein loading.

**(2) 12(S)HETE Measurements**

For assessment of 12(S)HETE, sciatic nerve and spinal cord samples were homogenized on ice in 15 mM Tris-HCI buffer (1 : 100 w/v) containing 140 mM NaCl, pH 7.6. Homogenates were centrifuged at 14,000 g (4°C, 20 minutes). 12(S)HETE concentrations were measured with the 12(S)-hydroxyeicosatetraenoic acid Enzyme Immuno Assay kit (Assay Designs, Ann Arbor, MI), in accordance with the manufacturer's instructions.

### 2.5. Statistical Analysis

The results are expressed as Mean ± SEM. Data were subjected to equality of variance *F* test and then to log transformation, if necessary, before one-way analysis of variance. Where overall significance (*P* < .05) was attained, individual between-group comparisons for multiple groups were made using the Student-Newman-Keuls multiple range test. When between-group variance differences could not be normalized by log transformation (datasets for body weights and plasma glucose), the data were analyzed by the nonparametric Kruskal-Wallis one-way analysis of variance, followed by the Bonferroni/Dunn test for multiple comparisons. Individual pair-wise comparisons between the groups fed NC and HFD (16-wk time point) and between the corresponding groups before (16-wk time point) and after (23-wk time point) PMI-5011 treatment were made using the unpaired or paired two-tailed Student's *t*-test or Mann-Whitney rank sum test where appropriate. Significance was defined at *P* ≤ .05.

## 3. Results

A 16-week HFD feeding resulted in 40% difference in body weights between HFD-fed mice and those fed NC (Table 1). Similar (39%) differences between the two groups maintained at the end of the study. A 7-week PMI-5011 treatment did not affect body weights in either NC- or HFD-fed mice. A 16-week HFD feeding resulted in a modest (14.5%) increase in non-fasting blood glucose concentrations compared with the mice fed NC. The difference between the two groups was 17% at end of the study. PMI-5011 reduced non-fasting blood glucose concentration in HFD-fed mice by 8.9%, compared with the baseline level (16-wk time point) in the same group (*P* < .05), without affecting hyperglycemia in the NC-fed group. 

HFD-fed mice displayed clearly manifest MNCV and SNCV deficits at both 16- and 23-week time points compared with the mice fed NC (Table 2). Note that both MNCV and SNCV were similar in NC-fed mice at the beginning of the study and at the 16-wk time point which indicates that HFD feeding-induced nerve conduction slowing did not develop due to affected peripheral nerve growth and maturation. PMI-5011 treatment essentially normalized MNCV and reduced SNCV deficit (to 6% versus 14% at baseline) in HFD-fed mice. PMI-5011 did not affect either MNCV or SNCV in the NC-fed group. 

Both 16- and 23-week HFD feedings resulted in a clearly manifest thermal hypoalgesia detected by both plantar and tail-flick tests (Table 3). Paw withdrawal and tail-flick response latencies were 59% and 86% higher (a 16-week time point) and 73% and 78% higher (a 23-week time point) in HFD-fed mice compared with the corresponding NC-fed groups. PMI-5011 treatment alleviated thermal hypoalgesia in HFD-fed mice, without affecting paw withdrawal or tail-flick response latencies in the NC-fed group. Another sensory abnormality developing in HFD-fed mice was tactile allodynia. Tactile withdrawal thresholds in response to light touch with flexible von Frey filaments were reduced by 38% and 50% after HFD feeding for 16 and 23 weeks, respectively. PMI-5011 treatment alleviated, although did not completely correct, tactile allodynia in HFD-fed mice, without increasing tactile response thresholds in the NC-fed group. Mechanical withdrawal thresholds were 25% higher (a 16-week time point) and 35% higher (a 23-week time point) in HFD-fed mice compared with the corresponding NC-fed groups, consistent with the development of mechanical hypoalgesia. PMI-5011 treatment alleviated mechanical hypoalgesia in HFD-fed mice, without affecting mechanical withdrawal thresholds in the NC-fed group.

**Figure 1:**
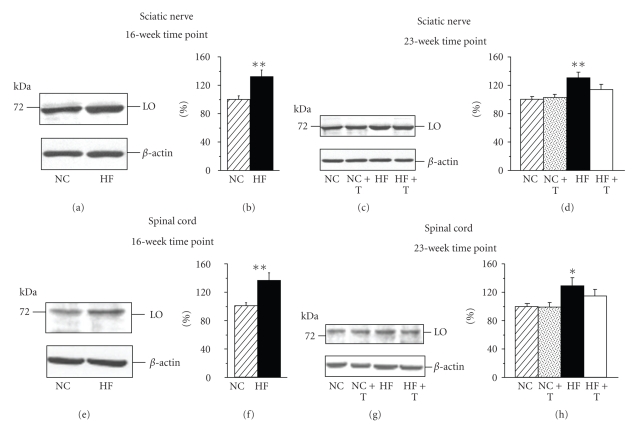
Representative Western blot analyses ((a), (c), (e), (g)) and content (densitometry, ((b), (d), (f), (h))) of mouse sciatic nerve ((a)–(d)) and spinal cord ((e)–(h)) 12/15-lipoxygenase expressions after 16-week ((a), (b) and (e), (f)) and 23-week ((c), (d) and (g), (h)) feedings with normal chow or high-fat diet with or without PMI-5011. NC: normal chow, HF: high fat diet, T: treatment. Mean ± SEM, *n* = 7-8 per group. *^,^**P < .05 and < .01 versus mice fed NC.

Sciatic nerve and spinal cord LO expressions were increased by 32% and 36% (a 16-week time point) and 31% and 29% (a 23-week time point) in HFD-fed mice compared with those fed NC (Figure 1). PMI-5011 did not affect LO expression in either tissue in the NC-fed mice. The extract tended to reduce LO expression in sciatic nerve and spinal cord of HFD-fed mice, but the differences with the corresponding baseline values did not achieve statistical significance.

**Figure 2:**
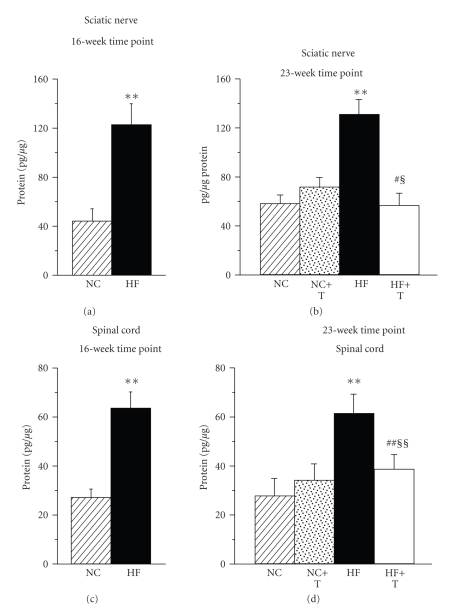
12(S)-hydroxyeicosatetraenoic acid concentrations in sciatic nerve ((a), (b)) and spinal cord ((c), (d)) after 16-week ((a) and (c)) and 23-week ((b) and (d)) feedings with normal chow or high-fat diet with or without PMI-5011. NC: normal chow, HF: high fat diet, T: treatment. Mean ± SEM, *n* = 9–13 per group. **P < .01 versus mice fed NC; ^§,§§^ *P* < .05 and < .01 versus the baseline value (16-wk time point); ^£^ *P* < .05 versus mice fed NC (16-wk time point); ^#,##^ *P* < .05 and < .01 versus untreated mice fed HFD (23-wk time point).

Sciatic nerve and spinal cord 12(S)-HETE concentrations were increased by 176% and 136% (a 16-week time point) and 122% and 126% (a 23-week time point) in HFD-fed mice compared with the corresponding groups fed NC (Figure 2). PMI-5011 did not affect 12(S)-HETE concentrations in either tissue in the NC-fed mice, but reduced (sciatic nerve) and essentially normalized (spinal cord) this variable in HFD-fed mice.

**Figure 3:**
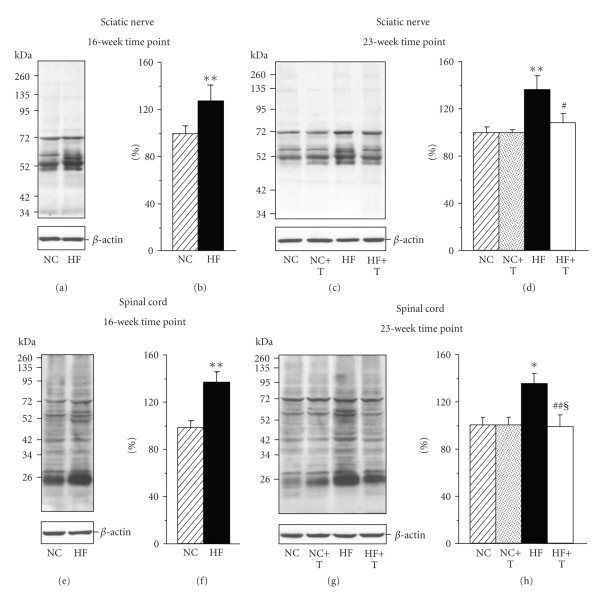
Representative Western blot analyses ((a), (c), (e), (g)) and content (densitometry, ((b), (d), (f), (h))) of mouse sciatic nerve ((a)–(d)) and spinal cord ((e)–(h)) nitrated protein expressions after 16-week ((a), (b) and (e), (f)) and 23-week ((c), (d) and (g), (h)) feedings with normal chow or high-fat diet with or without PMI-5011. NC: normal chow, HF: high fat diet, T: treatment. Mean±SEM, *n* = 7-8 per group. *^,^**P < .05 and < .01 versus mice fed NC; ^§^ *P* < .05 versus the baseline value (16-wk time point); ^#,##^ *P* < .05 and < .01 versus mice fed HFD.

Sciatic nerve and spinal cord nitrated protein expressions were increased by 32% and 40% (a 16-week time point) and 36% and 35% (a 23-week time point) in HFD-fed mice compared with the corresponding groups fed NC (Figure 3). PMI-5011 decreased spinal cord nitrated protein accumulation by 29%, and tended to reduce this variable in sciatic nerve although the difference with the baseline value was not of statistical significance. Statistically significant differences in both sciatic nerve and spinal cord nitrated protein levels were observed between HFD-fed groups maintained with and without PM-5011 treatment at the end of the study. The extract did not affect nitrated protein expression in either tissue in NC-fed mice.

## 4. Discussion

Peripheral neuropathy in human subjects with diabetes mellitus is typically characterized by motor and sensory nerve conduction slowing and increased vibration and thermal perception thresholds [1]. A higher prevalence of peripheral neuropathy and, predominantly, small sensory fiber neuropathy has been reported in subjects with metabolic syndrome, a condition that often includes prediabetes and obesity, and impaired glucose tolerance [2–4]. The present study provides evidence of the therapeutic efficacy of an ethanolic extract of *Artemisia dracunculus L.* on MNCV and SNCV deficits, thermal and mechanical hypoalgesia, and tactile allodynia in the model of peripheral neuropathy associated with prediabetes and alimentary obesity. The beneficial effects of PMI-5011 may at least partially be related to inhibition of oxidative-nitrosative stress and LO upregulation in peripheral nerve and spinal cord. Also note, that whereas the aforementioned findings have been obtained in peripheral nerve *in toto*, it is plausible that PMI-5011 inhibits both mechanisms in *vasa nervorum*. Endothelial cells contain LO and accumulate 12(S)HETE in response to high glucose [25, 26]. Both oxidative-stress [27, 28] and increased LO activity [25, 26] have been implicated in endothelial dysfunction, an important factor in MNCV and SNCV deficits associated with both diabetic [19, 20, 22, 24, 27–29] and prediabetic [5] neuropathy. 

Previous extensive studies in animal models revealed that MNCV and SNCV deficits and small sensory nerve fiber dysfunction are also amenable to treatment with numerous pharmacological agents. Electrophysiology remains a “gold standard” approach toward the diagnosis of peripheral diabetic neuropathy [1], and MNCV and SNCV have been assessed in the vast majority of experimental studies of potential new therapeutics as well as in all major clinical neuropathy trials [30–35]. Note that whereas experimental studies demonstrated a complete reversal of diabetes-induced MNCV and SNCV deficits by a variety of pharmacological agents (reviewed in [36, 37]), the results of clinical trials for example, those of aldose reductase and protein kinase C inhibitors, were quite modest [30, 31, 34, 35]. Such modest efficacy was most likely related to insufficient dosage, with test doses in clinical trials at least one order of magnitude lower than in the corresponding animal studies, and the use of higher doses was not possible in humans because of adverse side effects. Liver toxicity was a major cause of withdrawal of several aldose reductase inhibitors [38]. A significant proportion of patients in the recent 6-month study of the protein kinase C inhibitor ruboxistaurin displayed treatment-emergent adverse events [39]. Therefore, undoubtedly, the development of nonpharmacological and, in particular, complementary and alternative medicinal therapies for correction of diabetes-associated nerve conduction velocity deficits is of great importance. In the present study, the botanical extract PMI-5011 essentially reversed MNCV and SNCV deficits associated with prediabetic neuropathy. It also reduced thermal and mechanical hypoalgesia, that is, sensory loss, which is a major cause of foot ulceration and amputation in human subjects with diabetes mellitus [1]. Several herbal extracts and other products have been reported to alleviate diabetic neuropathic pain and sensory loss in diabetic rats and mice [40, 41]. However, to our knowledge, only primrose oil reversed MNCV and SNCV deficits as effectively [42] as it was observed in our study with PMI-5011.

As one can see from Table 2, PMI-5011 displayed minor hypoglycemic activity when administered to HFD-fed mice. However, it is highly unlikely that small (~12%–17%) differences in blood glucose concentrations were responsible for significant differences in nerve conduction velocities and variables of sensory neuropathy among NC-fed, HFD-fed, and PMI-5011-treated HFD-fed groups. This conclusion is in line with our previous observations in HFD-fed female mice which displayed neuropathic changes at the stage of impaired glucose tolerance, prior to development of overt hyperglycemia [6]. It is also in agreement with our recent studies in STZ-diabetic mouse model in which PMI-5011 did not affect hyperglycemia, but, nevertheless, alleviated functional manifestations of peripheral neuropathy (P. Watcho, R. Stavniichuk, I. G. Obrosova, unpubished). 

Evidence for the importance of oxidative-nitrosative stress in peripheral diabetic neuropathy is emerging, and several antioxidants [27, 28, 43–46], including peroxynitrite decomposition catalysts [44–46], have been found to counteract MNCV and SNCV deficits and small sensory nerve fiber dysfunction. HFD-fed mice display clearly manifest nitrosative stress in PNS [6], and the present study, and PMI-5011 essentially normalized nitrated protein content in both peripheral nerve and spinal cord. Therefore, beneficial effects of PMI-5011 on prediabetic neuropathy may at least partially be explained by antioxidant properties of the extract. 

Oxidative stress is closely linked to upregulation of 12/15-LO, an enzyme converting arachidonic acid to 12(S)-HETE, 15(S)-HETE, and a number of derivatives of these acids. These lipid-like compounds undergo spontaneous lipid peroxidation, which leads to induction of oxidative-nitrosative stress, activation of mitogen-activated protein kinases (MAPKs), and proinflammatory response [25, 26]. MAPK activation has been demonstrated to play an important role in peripheral diabetic neuropathy [47, 48]; furthermore, this phenomenon has been identified in human diabetic nerve [47]. Evidence for the importance of low-grade inflammation in diabetic neuropathy is also emerging from both experimental and clinical studies [49–53]. In a recent study, LO gene deficiency prevented western diet-induced increase in macrophage numbers and monocyte chemoattractant protein-1 overexpression in mouse visceral fat [54], thus directly implicating LO in HFD-induced inflammation. As we demonstrated previously [6] and in the current study, 12/15-lipoxygenase protein overexpression and activation are present in PNS at the prediabetic stage, prior to development of overt hyperglycemia. PMI-5011 treatment tended to inhibit HFD-induced 12/15-lipoxygenase overexpression in sciatic nerve, and significantly reduced 12(S)-HETE concentrations in both peripheral nerve and spinal cord. Thus, PMI-5011 may affect multiple mechanisms implicated in prediabetic neuropathic changes by inhibiting LO upregulation. It may also exert beneficial effect due to aldose reductase inhibiting properties [18]. Increased aldose reductase activity in tissue-sites for diabetic complications, including diabetic peripheral nerve, is known to contribute to the formation of advanced glycation end products, oxidative-nitrosative stress, and protein kinase C and poly(ADP-ribose) polymerase activations [22, 23, 55–57]. All these mechanisms have been implicated in the pathogenesis of peripheral diabetic neuropathy (reviewed in [37]). In addition, increased AR activity may cause LO overexpression and activation by promoting activation of nuclear factor-*κ*B and activator protein-1 as well as cytosolic Ca^++^ accumulation (reviewed in [55]), that is, via upregulation of three factors essentially required for LO gene expression and activity [25, 26]. 

In conclusion, the ethanolic extract of *Artemisia dracunculus L.*, PMI-5011, alleviates peripheral nerve dysfunction in neuropathy associated with prediabetes and alimentary obesity, potentially, by multiple mechanisms that are likely to include, but are not limited by, inhibition of oxidative-nitrosative stress and LO activation. PMI-5011, a safe and nontoxic product, may find use in management of clinical diabetic neuropathy at the earliest stage of disease. Further studies are needed to determine whether PMI-5011 treatment is effective against functional and structural deficits of advanced diabetic neuropathy.

## Figures and Tables

**Table 1:** Body weights and non-fasting blood glucose concentrations in normal chow and high fat diet-fed mice maintained with and without PMI-5011 treatment.

Variable	Body weight, g		Blood glucose, mmol l^−1^	
Group	Before T (16-wk time point)	After T (23-wk time point)	Before T (16-wk time point)	After T (23-wk time point)
NC	37.5 ± 0.57	36.8 ± 0.74	7.50 ± 0.19	7.67 ± 0.25
NC + T	36.9 ± 0.80	36.3 ± 0.74	7.28 ± 0.18	7.82 ± 0.31
HFD	52.6 ± 0.36**	51.3 ± 0.57**	8.58 ± 0.22**	8.95 ± 0.50**
HFD + T	53.7 ± 0.53**	50.8 ± 0.90**	8.59 ± 0.19**	7.82 ± 0.33^§,##^

Data are expressed as Mean ± SEM, *n* = 10–33 per group. NC: normal chow; T: treatment; HFD: high fat diet; *^,^**P < .05 and < .01 versus mice fed NC; ^§^ *P* < .05 versus the baseline value (16-wk time point); ^##^ *P* < .01 versus untreated mice fed HFD (23-wk time point).

**Table 2:** Motor and sensory nerve conduction velocities in normal chow and high fat diet-fed mice maintained with and without PMI-5011 treatment.

Groups	NC	NC + T	HFD	HFD + T
Variables				
Baseline (prior to the beginning of HFD feeding)				

MNCV, ms^−1^	50.8 ± 0.75			
SNCV, ms^−1^	38.4 ± 0.71			

16-wk time point (prior to PMI-5011 treatment)				

MNCV, ms^−1^	51.8 ± 0.87		46.3 ± 0.77**	
SNCV, ms^−1^	39.1 ± 0.65		33.8 ± 0.81**	

23-wk time point (final measurements)				

MNCV, ms^−1^	51.4 ± 1.78	52.3 ± 2.14	45.9 ± 1.18*	51.0 ± 1.45^§,#^
SNCV, ms^−1^	38.7 ± 0.92	37.3 ± 1.08	33.6 ± 1.14**	36.8 ± 0.92^§,£,#^

Data are expressed as Mean ± SEM, *n* = 10–12 per group. NC: normal chow; T: treatment; HFD: high fat diet; MNCV: motor nerve conduction velocity; SNCV: sensory nerve conduction velocity. *^,^**P < .05 and < .01 versus mice fed NC; ^§^ *P* < .05 versus the baseline value (16-wk time point); ^£^ *P* < .05 versus mice fed NC (16-wk time point); ^#^ *P* < .05 versus untreated mice fed HFD (23-wk time point).

**Table 3:** Variables of small sensory fiber neuropathy in normal chow and high fat diet-fed mice maintained with and without PMI-5011 treatment.

Groups	NC	NC + T	HFD	HFD + T
Variables				
Baseline (prior to the beginning of HFD feeding)				

Paw withdrawal latency, s^−1^	8.8 ± 0.15			
Tail flick response latency, s^−1^	2.41 ± 0.08			
Tactile withdrawal threshold, g	1.76 ± 0.12			
Mechanical withdrawal threshold, g	11.5 ± 0.31			

16-wk time point (prior to PMI-5011 treatment)				

Paw withdrawal latency, s^−1^	8.2 ± 0.17		13.0 ± 0.31**	
Tail flick response latency, s^−1^	2.48 ± 0.09		4.62 ± 0.13**	
Tactile withdrawal threshold, g	1.72 ± 0.13		1.06 ± 0.09**	
Mechanical withdrawal threshold, g	10.6 ± 0.28		13.3 ± 0.24**	

23-wk time point (final measurements)				

Paw withdrawal latency, s^−1^	9.1 ± 0.32	10.3 ± 0.41	15.7 ± 1.14**	11.4 ± 0.68^§,∗,##^
Tail flick response latency, s^−1^	2.29 ± 0.11	2.52 ± 0.10	4.07 ± 0.15**	2.59 ± 0.10^§§,##^
Tactile withdrawal threshold, g	1.52 ± 0.21	1.45 ± 0.16	0.76 ± 0.03**	1.20 ± 0.08^££,#^
Mechanical withdrawal threshold, g	11.2 ± 0.46	11.2 ± 0.36	15.2 ± 0.10**	12.3 ± 0.59^£,##^

Data are expressed as Mean ± SEM, *n* = 10–12 per group. NC: normal chow; T: treatment; HFD: high fat diet; *^,^**P < .05 and < .01 versus corresponding controls; ^§,§§^ *P* < .05 and < .01 versus the baseline value (16-wk time point); ^£,££^ *P* < .05 and < .01 versus mice fed NC (16-wk time point); ^#,##^ *P* < .05 and < .01 versus untreated mice fed HFD (23-wk time point).
